# Amplifying Women's Voices in Menopause Research: The Importance of Inclusive Perspectives

**DOI:** 10.1111/hex.70190

**Published:** 2025-02-17

**Authors:** Chunyu Zhang, Xiaohan Sun, Bei Li

**Affiliations:** ^1^ School of Economics and Management Guangxi Normal University Guilin China; ^2^ Key Laboratory of Digital Empowerment Economic Development (Guangxi Normal University) Education Department of Guangxi Zhuang Autonomous Region Guilin China

Dear Editor,

I recently read the article titled ‘Amplifying Women's Voices in Menopause Research: The Importance of Inclusive Perspectives’ published in *Health Expectations*. I am writing to commend the insightful work of Cronin et al. This study provides a valuable contribution by offering a more comprehensive understanding of the factors influencing women's menopausal experiences and emphasising the critical importance of women's participation in research. The findings underscore that incorporating women's voices into research and policymaking is essential for improving their well‐being [1].

While this study offers valuable insights, it also has certain limitations. First, it lacks specific examples or case studies to illustrate the importance of an inclusive perspective. Additionally, the study relies predominantly on qualitative analysis and insights, with limited quantitative data to support its claims. Finally, although it suggests using surveys, focus groups, and participatory studies for data collection, the research does not detail the implementation steps for these methods or the data analysis process. This lack of detail makes it difficult for readers to assess the reliability and validity of the findings. Addressing these gaps in future research could significantly enhance the study's robustness and accuracy.

To strengthen the persuasiveness of the research, I propose the following suggestions:

1. Incorporating detailed case studies or examples of successful inclusive research projects on menopause. This would not only demonstrate the practical impact of inclusion research but also provide actionable guidance for researchers and policymakers.
2. Exploring challenges to incorporating women's voices and developing strategies to overcome these barriers. Drawing on the literature and personal experiences could offer nuanced insights into this aspect.
3. Expanding the scope to investigate quantitative data on women's menopause experiences and evaluating the effectiveness of inclusion research methods across different regions. Combining quantitative data with case studies would provide a stronger evidence base and theoretical foundation. This would not only enhance the accuracy of findings but also increase their credibility and relevance for application in practice [2].

I appreciate the opportunity to share my thoughts and commend Cronin et al. for their impactful contribution. I hope these suggestions will inspire further research to advance understanding and advocacy in this critical area.

## Author Contributions

**Chunyu Zhang:** conceptualisation, methodology, supervision, funding acquisition, project administration, resources, writing – review and editing. **Xiaohan Sun:** writing – original draft. **Bei Li:** writing – review and editing.

## Ethics Statement

This study was exempted by the Ethics Committee of the School of Economics and Management, Guangxi Normal University.

## Conflicts of Interest

The authors declare no conflicts of interest.

## Data Availability

This study did not generate any datasets.
